# *Plasmodium* species differentiation by non-expert on-line volunteers for remote malaria field diagnosis

**DOI:** 10.1186/s12936-018-2194-8

**Published:** 2018-01-30

**Authors:** Alejandra Ortiz-Ruiz, María Postigo, Sara Gil-Casanova, Daniel Cuadrado, José M. Bautista, José Miguel Rubio, Miguel Luengo-Oroz, María Linares

**Affiliations:** 1 Research Institute Hospital 12 de Octubre, Universidad Complutense de Madrid, Ciudad Universitaria, 28040 Madrid, Spain; 2 Biomedical Image Technologies Group, DIE, ETSI Telecomunicación, Universidad Politécnica de Madrid, CEI Moncloa UPM-UCM, Madrid, Spain; 3 Malaria and Emerging Parasitic Diseases Laboratory, National Microbiology Centre, Instituto de Salud Carlos III, Madrid, Spain; 4 Centro de Investigación Biomédica en Red en Bioingeniería, Biomateriales y Nanomedicina (CIBER-BBN), Madrid, Spain; 5 SPOTLAB, S.L. C/Gran Vía 39, 2º, 28013 Madrid, Spain

**Keywords:** Malaria species identification, Crowdsourcing, Remote diagnosis

## Abstract

**Background:**

Routine field diagnosis of malaria is a considerable challenge in rural and low resources endemic areas mainly due to lack of personnel, training and sample processing capacity. In addition, differential diagnosis of *Plasmodium* species has a high level of misdiagnosis. Real time remote microscopical diagnosis through on-line crowdsourcing platforms could be converted into an agile network to support diagnosis-based treatment and malaria control in low resources areas. This study explores whether accurate *Plasmodium* species identification—a critical step during the diagnosis protocol in order to choose the appropriate medication—is possible through the information provided by non-trained on-line volunteers.

**Methods:**

88 volunteers have performed a series of questionnaires over 110 images to differentiate species (*Plasmodium falciparum, Plasmodium ovale, Plasmodium vivax, Plasmodium malariae, Plasmodium knowlesi*) and parasite staging from thin blood smear images digitalized with a smartphone camera adapted to the ocular of a conventional light microscope. Visual cues evaluated in the surveys include texture and colour, parasite shape and red blood size.

**Results:**

On-line volunteers are able to discriminate *Plasmodium* species (*P. falciparum*, *P. malariae, P. vivax*, *P. ovale*, *P. knowlesi*) and stages in thin-blood smears according to visual cues observed on digitalized images of parasitized red blood cells. Friendly textual descriptions of the visual cues and specialized malaria terminology is key for volunteers learning and efficiency.

**Conclusions:**

On-line volunteers with short-training are able to differentiate malaria parasite species and parasite stages from digitalized thin smears based on simple visual cues (shape, size, texture and colour). While the accuracy of a single on-line expert is far from perfect, a single parasite classification obtained by combining the opinions of multiple on-line volunteers over the same smear, could improve accuracy and reliability of *Plasmodium* species identification in remote malaria diagnosis.

**Electronic supplementary material:**

The online version of this article (10.1186/s12936-018-2194-8) contains supplementary material, which is available to authorized users.

## Background

Despite the efforts made in malaria control during the last years, malaria is still the most devastating parasitic disease in tropical and subtropical areas, that causes approximately 500,000 deaths as reported by the World Health Organization (WHO) [1]. Although most of deaths are caused by *Plasmodium falciparum*, it is important to highlight an increase of the relative contribution of the non-falciparum *Plasmodium* species to the global malaria burden as the incidence of *P. falciparum* falls [2]. Two other species are also capable of causing severe disease: *Plasmodium vivax*, responsible for half of the world’s malaria cases outside Africa [3, 4]; and *Plasmodium knowlesi*, the most common cause of human malaria in Malaysia and increasing in other Southeast Asian countries [2, 3]. *Plasmodium malariae* is a slow-growing parasite with a wide geographic distribution. Although generally regarded as a benign cause of malaria, it has been associated with nephrotic syndrome, particularly in young children, and can persist in the host for years [5]. Finally, *Plasmodium ovale* distribution is highest in sub-Saharan Africa, but its prevalence is deemed to be lower compared to *P. malariae* [6].

An accurate classification of the species at the time of diagnosis is crucial for directing people to an appropriate treatment, preventing over-treatment and injudicious use of anti-malarials, especially in co-endemic areas [2, 6–8]. Malaria species identification is particularly important when different species have separate treatment policies, most commonly artemisinin-based combination therapy (ACT) for *P. falciparum* (and *P. vivax* at regions where chloroquine resistance is prevalent) and chloroquine for non-falciparum species. Moreover, diagnosis of *P. vivax* and *P. ovale* is still required to allow administration of anti-hypnozoite treatment to prevent relapses. In areas which are also endemic for *P. knowlesi*, an accurate diagnosis is important to reach an epidemiological surveillance of this potentially fatal emerging zoonotic infection [2, 9].

Since 2010, WHO has recommended that all persons with suspected malaria in all settings should undergo malaria diagnostic testing, by either microscopy or rapid diagnostic test (RDT) [3]. As RDT shows poor or very limited sensitivity for detecting some *Plasmodium* species [9], the examination of Giemsa-stained thick and thin films of blood by light microscopy from malaria patients, remains the gold standard for malaria diagnosis in most parts of the malaria-endemic world [2, 8, 10]. This enables to obtain parameters such as parasite detection, estimation of parasite densities and identification of *Plasmodium* species. Accurate identification of the infecting *Plasmodium* species relies on detailed examination of parasite morphological characteristics such as size, shape, pigmentation, besides the size and shape of the parasitized red blood cells (RBC) and the inclusions therein [6]. However, this process requires trained technicians to efficiently and accurately detect parasite and classify its species. With other factors such as some technical limitations and potential human inconsistency [11–13], the detection and classification can be further degraded [8]. Previous research [6] has shown that laboratory personnel involved in microscopic diagnosis of malaria might experience difficulties in differentiating *Plasmodium* infections, which suggest that personnel is not always well trained in non-falciparum infections in endemic areas where two or more species are present. Moreover, health facilities at high season might receive hundreds of people and become impossible to assist patients with a correct diagnosis [2], because of the time required for obtaining an accurate diagnosis even for the experts [11].

In the last years, several methodologies based on crowdsourcing tools have been developed to solve big data challenges [14]. Games with a specific purpose generate strategies to incentive people to solve problems, a minimum contribution translates in a huge impact [15]. Systems based on crowdsourcing tools have been applied to solve challenges such as public health problems [12, 16–18].

The project MalariaSpot has used crowdsourcing to achieve a quantification of malaria parasites in thick blood smears [19]. The results of the use of this platform showed that, on average, the combination of 22 gamers or more, regardless of the player’s experience, was enough to obtain almost perfect parasite counting (99%) in the tested images [17, 19]. Showing that the application of these new technologies in disease diagnosis could play a great role improving the correct treatment at low resource areas [20–22].

The present report shows the potential of a digital platform based on crowdsourcing techniques to train non-experts in order to classify *Plasmodium* species causing malaria in humans. This low-cost tool could assist a quick diagnosis system providing correct and rapid identification of the malaria species. On this basis, the accuracy and effectiveness of gamers to diagnose Giemsa-stained malaria thin films was evaluated, referred to the five *Plasmodium* species and digitalized by a smartphone coupled to a conventional light microscopy. The results of this work showed that it is possible to obtain an accurate diagnosis of the species by aggregation of non-expert answers about simple visual cues: texture and colour patters, the shape and the size of the parasitized erythrocyte.

## Methods

### Digitalization of clinical samples

The malaria slides used in this study were obtained from the Malaria and Emerging Parasitic Diseases Laboratory (Spanish National Biobanks Registry Nº: C.0001392). All slides were previously re-examined and verified by expert microscopists, as well as confirmed by PCR to give a species-specific diagnosis.

Twenty Giemsa stained anonymous thin blood smears were digitalized using a mobile phone (Sony Xperia Z2) coupled to an adaptor (Universal Digiscoping, Cellscope). Images were acquired with the “Camera FV-5 Pro” mobile application, under microscope (Zeiss AX05COP2), using an oil immersion objective (1000× magnification). The images were captured in the PNG format, with a resolution of 3.1 Mpx. To achieve an optimum exposure in the images acquired, the steps recommended by the CDC were followed.

A total of 110 images were chosen from this image repository, according to pre-established requirements like the proportion of appearance of the distinct stages in the different parasites as well as those images which represent with more confidence the species-specific parameters and taking out of consideration those samples with presence of mix infections, bad staining and or highly deteriorated.

### Study design

**Fig. 1:**
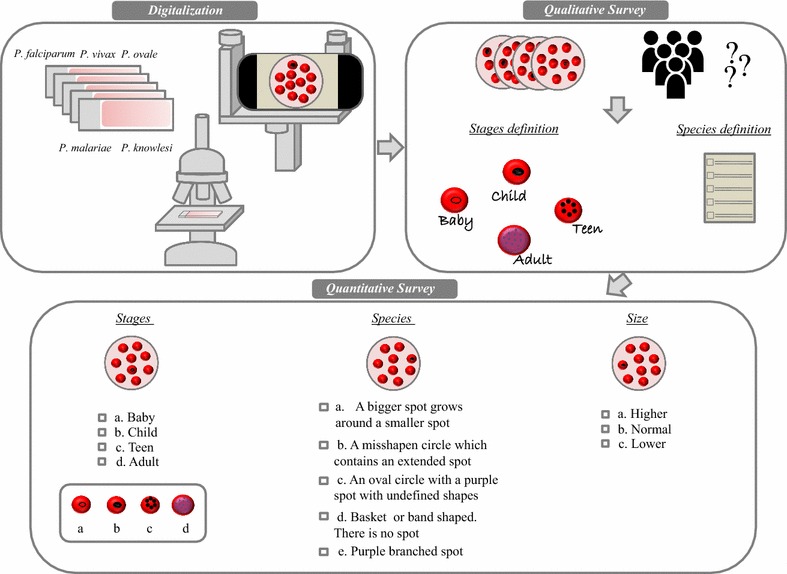
Scheme showing the study design. First, thin blood smears were digitalized using a mobile coupled to an adaptor (Universal Digiscoping, Cellscope). In a qualitative survey, images were shown to volunteers in order to find an easier and colloquial way to designate the different stages and species of *Plasmodium*. Using the definitions suggested by non-experts, a quantitative form was designed to evaluate the ability of new participants to provide an accurate classification of *Plasmodium* species, by differentiating the three main parameters observed on parasitized red blood cells: parasite stage, parasite shape and infected red blood cell size

In order to evaluate the accuracy and effectiveness of on-line volunteers to diagnose Giemsa-stained malaria thin films, a quantitative and qualitative approach were assessed through two types of questionnaires (Fig. 1).

#### Qualitative survey

Due to the difficulty of the malaria terminology for a non-expert volunteer, this first “qualitative survey” was an oral interview to understand the type of terminology and visual cues that could be efficient non-expert learning. The set of 110 representative images of the five parasite species at the different stages was shown to six initial volunteers (not involved in the rest of the surveys and without any biomedical knowledge). Three groups of questions were established; the first group consisted of a set of 50 reduced images showing just the infected red blood cell (15 young rings, 10 trophozoites, 10 schizonts and 10 gametocytes) and the volunteers were asked about the differences among the stages and how they could define them in one word. The second group was composed of a set of forty images showing a microscopic field of view (two for each stage and species) asking for a representative short definition about the five species at each stage. Twenty additional images were shown to the volunteers in order to validate their ability to recognize the parasites.

#### Quantitative survey

A main survey was elaborated to determine the ability on non-experts to recognize the different malaria parasites species and their stages with 53 non-expert volunteers. A follow up questionnaire was also designed to determine ability to differentiate changes in the RBC shape size among the five *Plasmodium* species in 29 volunteers.

Prior to the main survey, the convenience of using a “supporting example image” that shows a visual example of the different species that the volunteers need to classify was assessed with a group of 32 volunteers. This survey was created using twelve images of the four different life-cycle stages: six, in which each image was shown with the supporting example image mentioned above and other six in which each image was shown alone to the same group of volunteers. For each image, the volunteer chose one of these four responses referred to the parasite: *“baby”* for young trophozoites*, “child”* for mature trophozoites*, “teen”* for schizonts and *“adult”* for gametocytes, according to the definitions obtained after the results of the qualitative study. After analysing the results, the supporting example image was included in the main survey (Additional file 1).

A unique form was used in the main survey to study the ability of volunteers to differentiate the stages and the species. For stages, a set of twenty-two questions were presented, including a strip of images as a supporting media, which consisted in an example image from each parasite species at each parasite stage. Thus, each volunteer could choose one out four words (“*baby”, “child”, “teen” and “adult”*) corresponding with the life-cycle stage.

The part of the survey referred to the species presented a set of forty-one questions. In this case, each volunteer chose one out five definitions corresponding to each malaria parasite. These definitions were formulated with the help of the “qualitative survey” mentioned above. The same proportion as the different stage-forms appear in real samples was maintained.

Finally, as a follow up questionnaire and to assess whether non-experts were able to differentiate the size of RBC infected by the different species, a separately form was designed using a set of forty-one questions. For each infected RBC, volunteers chose between “higher”, “normal” or “lower” size.

In order to assign a probability value of diagnosis to each image, the results which obtained the higher percentage of answers (and higher than the one expected randomly) for the questionnaires about species and size were combined.

### Statistical analysis

All statistical analyses were performed using the GraphPad Prism Software (version 6). The parametric T-Student test or the non-parametric test U-Mann–Whitney was used to compare between two groups and the parametric ANOVA Test or the non-parametric test Kruskal–Wallis was used when more of two groups were compared. Differences between means were considered significant when the *P* value was ≤ 0.05.

## Results

### Description of the different species and stages by non-expert observers

The analysis of the results regarding the qualitative survey suggests that an easier and colloquial way to designate the parasitic stages that make non-experts more comfortable and efficient is to establish a relationship between the stages of the parasite and the human ages: *“baby”* for young trophozoites*, “child”* for mature trophozoites*, “teen”* for schizonts *and “adult”* for gametocytes. The answers about the different species allowed to generate an easy, simple and characteristic definition of each of the stages of the malaria species (Table 1).

**Table 1:** Characteristic definition of each of the stages of the malaria species

	*P. falciparum*	*P. vivax*	*P. ovale*	*P. malariae*	*P. knowlesi*
Ring	Purple spot with a thin ring	Purple spot with deformed body	Ring with a large purple spot	Purple spot with a thick body	Purple spot (or spots) with an amorphous thick ring
Trophozoite	A bigger spot grows around a smaller spot	A misshapen circle which contains an extended spot	An oval circle (sometimes with small corners) which contains a purple spot with undefined shapes	Basket or band shaped. There is no spot	Purple branched spot (the branches are continuous or discontinuous)
Schizont	Not stablished	Not defined purple spots inside a circle	More than one spot (occasionally they are not very clear) inside an oval circle (sometimes with small corners)	Diffuse purple spots around a darker spot	Defined purple spots easy to count
Gametocyte	Banana/sausage shaped	Extended, big spot	Row of accumulated spots	A big stained spot which almost fill the circle	A big spot which contains small spots

Definitions were elaborated based on the answers of a small group of volunteers (n = 6) not involved in the rest of the surveys

### Non-expert participants are able to differentiate parasite stages

**Fig. 2:**
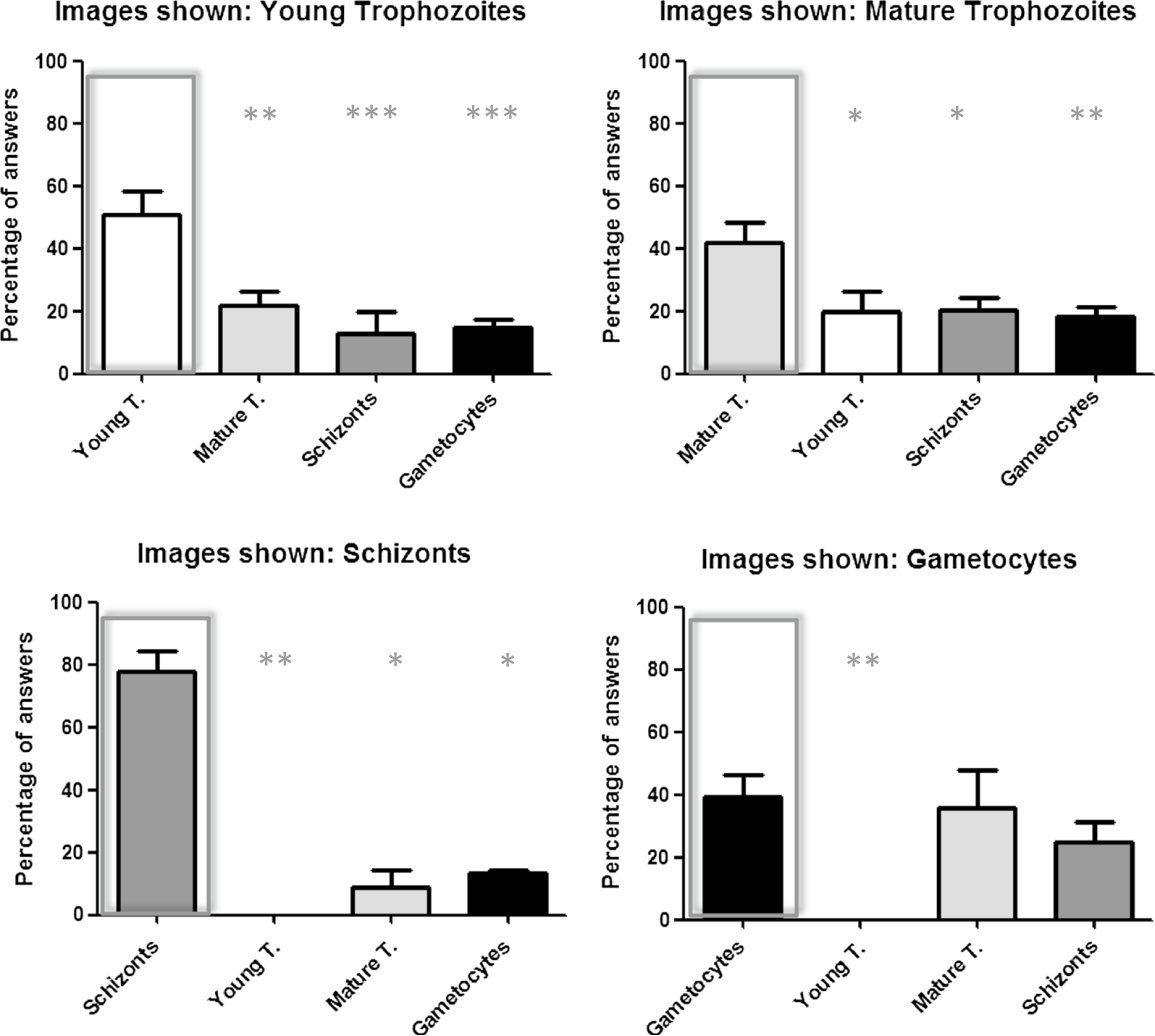
Percentage of answers obtained for the different stages shown. Correct answer is marked within a rectangle. Values given are the mean ± SEM calculated for the different images shown in a total of 53 volunteers. Asterisk indicates a significant difference between the percentage of response of the correct answer and each other three possibilities. *P < 0.05; **P < 0.01; **P < 0.005. Young trophozoites (young t.), mature trophozoites (mature t.)

*Plasmodium* identification frequently requires classifying the different morphological stages. The results obtained in the survey referred to the stages showed that most of the volunteers were able to identify the *Plasmodium* stage in the different images shown (Fig. 2). For all the stages, the percentage of answers for the correct form shown in the survey was higher than the expected randomly. Moreover, young and mature trophozoites, as well as schizonts, were significantly discriminated from all the other forms (young versus mature trophozoites, *P* < 0.05; young versus schizonts and gametocytes, P < 0.01; mature trophozoites versus schizonts, *P* < 0.05; mature trophozoites versus gametocytes, *P* < 0.01). Gametocytes were significantly differentiated from young trophozoites (*P* < 0.01) but not from mature trophozoites and schizonts.

### Non-expert participants are able to differentiate parasite species

Then, the ability of non-expert participants to differentiate the five *Plasmodium* species that affect human (*P. falciparum, P. vivax, P. ovale, P. malariae* and *P. knowlesi*) was evaluated. For this, different images of the parasite species were shown to volunteers in the survey.

**Fig. 3:**
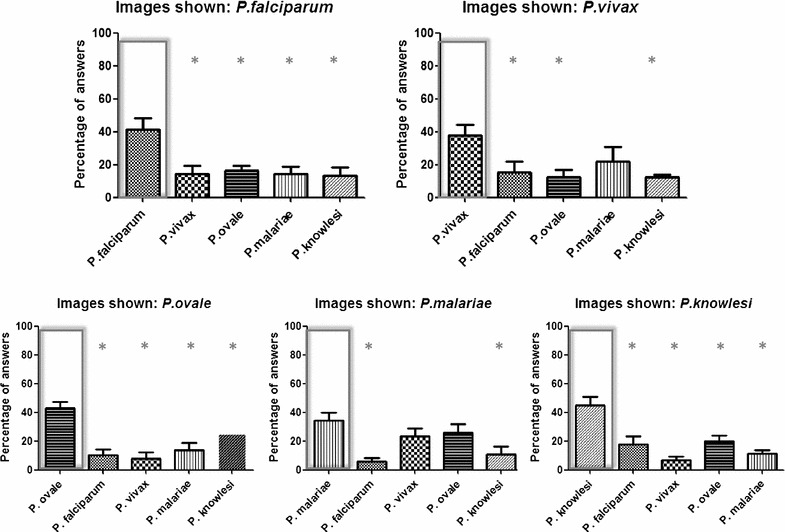
Percentage of answers obtained for the different species shown. Correct answer is marked within a rectangle. Values given are the mean ± SEM calculated for the different images shown in a total of 53 volunteers. Asterisk indicates a significant difference between the percentage of response of the correct answer and each of the other four possibilities. *P < 0.05

The results showed that, for all queries, the correct species shown in the survey received the highest percentage of answers (Fig. 3). *Plasmodium falciparum*, *P. ovale* and *P. knowlesi* were statistically discriminated from all the other species (*P* < 0.05). *Plasmodium vivax* was statistically differentiated from all the species with the exception of *P. malariae (P. vivax* versus *P. falciparum, P. ovale* and *P. knowlesi, P* < 0.05)*. Plasmodium malariae* was not distinguished significantly from *P. vivax* and *P. ovale (P. malariae* versus *P. falciparum* and *P. knowlesi, P* < 0.05).

**Fig. 4:**
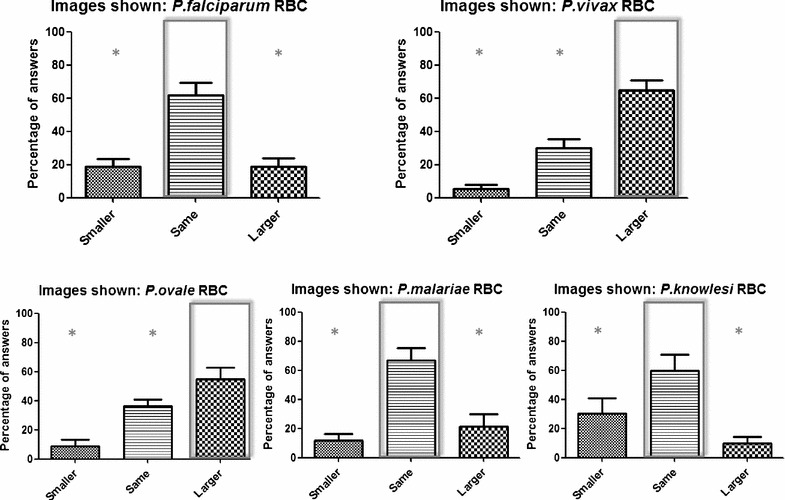
Percentage of answers obtained for the size of the different infected erythrocytes shown. Correct answer is marked within a rectangle. Values given are the mean ± SEM calculated for the different images shown in a total of 29 volunteers. Asterisk indicates a significant difference between the percentage of response of the correct answer and each of the other two possibilities. *P < 0.05. *RBC* red blood cells

Interestingly, in *P. malariae* infections, RBC are normal or smaller than normal size. In contrast, in *P. ovale* and *P. vivax* infections, RBC are often larger than the normal ones. Then, these species could be differentiated by the red blood cell size, a parameter that was not included in the survey. Therefore, a new experiment with a different set of non-expert participants inquiring about to RBC size of different *Plasmodium* species was performed. *Plasmodium ovale* and *P. vivax* RBC were significantly identified as “larger” (*P* < 0.05) and *P. malariae*, as well as *P. falciparum* and *P. knowlesi* were statistically identified as “same” size (*P* < 0.05) (Fig. 4).

Combined with the previous results, these data demonstrate the potential ability of non-expert volunteers to statistically differentiate the five *Plasmodium* species which infect humans. When a probability value of diagnosis was assigned to each image, based on the answers obtained about species and size, sensitivity of 90% was reached for *P. ovale*, 80% for *P. vivax* and *P. falciparum* and 70% for *P. malariae* and *P. knowlesi*. When *P. vivax* versus *P. falciparum* was compared, sensitivity reach scores of 80–100%.

## Discussion

According to the WHO an ideal test would be inexpensive, consistent, highly sensitive, adequately specific, quantitative and species-differentiating. Microscopy is the golden standard diagnosis method for malaria, and at the same time the cheapest, but it has the disadvantage that a high level of training and experience is needed to make a correct diagnosis especially for species identification. The present study explored the possibility of use the power of non-expert on-line volunteers to differentiate the five human *Plasmodium* species from samples of thin blood film digitalized by a smartphone coupled to a conventional light microscopy by an inexpensive way using a combination of microscopy and social networks.

Previously, the ability of non-expert volunteers to differentiate between infected or uninfected RBC has been analysed [23]. Nevertheless, to our knowledge, this is the first study that addresses species identification of the five parasites at the same time using an on-line platform.

Malaria species classification on thin blood films is very challenging, even for an experienced microscopist, and frequently requires to classify the different stages morphology [8]. To this regard, our work demonstrated that non-experts are able to classify the distinct intraerythrocytic forms. Comparing the shape of the different *Plasmodium* stages, volunteers were able to improve their performance by associating the cycle of the parasite with the human ages terms- easy to remember given that the training was in the order of minutes (“*baby*”, “*child*”, “*teen*” and “*adult*”). Furthermore, volunteers were more accurate when supporting example images were included at the moment of making the decision. Non-expert volunteers were able to differentiate the four parasite stages, although they presented difficulties to discriminate gametocytes from the other two mature forms (mature trophozoites and schizonts) in line with what have been previously reported in the literature for expert microscopists [22, 24, 25]. As gametocyte carriage substantially contributes to the human infective reservoir for onward transmission to mosquitoes [26, 27], it is important to find methodologies to overcome this limitation.

Non-experts were also able to discriminate *Plasmodium* species including *P. knowlesi*. It is well established that misdiagnosis of *P. knowlesi* with other species is frequent [14] and, under the microscope, it is frequently misdiagnosed with *P. malariae*, as late trophozoites are very similar, or even it is confounded with *P. falciparum*, as the young ring forms are similar [9, 28]. It should be noted that for other species, high false negative and positive results frequently suggest non-familiarity with the non-falciparum infections, which is reduced after training assessments [6]. The non-familiarity with the most recently introduced *P. knowlesi,* could also affects to the distinction, particularly between *P. falciparum, P. vivax* and *P. malariae* [29, 30]. Nevertheless, it is worthy to note that, although a better training in the endemic areas could improve classification, in the present study a high proportion of P. *knowlesi* rings were still confounded with *P. falciparum*, in the light with the previous reports.

In this work, volunteers experienced difficulties to distinguish *P. vivax* versus *P. malariae*, and this latter versus *P. ovale.* It has been previously reported that it is very hard to differentiate the ring form of *P. malariae* and *P. vivax* by microscopy since they are very similar [31]. In a separately study, microscopy gave an incorrect diagnosis in 1.5% of the positive cases, mistaking *P. vivax* with *P. ovale* and *P. malariae* [32].

Interestingly, these species could be differentiated by the red blood cell size, and because of that, this variable was analyzed in a separately survey. As expected, *P. ovale* and *P. vivax* RBC were significantly identified as “larger” than *P. malariae*, *P. falciparum* and *P. knowlesi* [33]. Combined with the previous results, data demonstrate the potential ability of non-expert volunteers to statistically differentiate all the five *Plasmodium* species.

Generally, this proof of concept demonstrates that non-expert volunteers can learn the tasks for image recognition of the different species, producing a consistent and sensitive classification of the different species when opinions from multiple volunteers over the same sample are aggregated. This methodology paves the way to create tele-microscopy systems that combine remote image digitalization, human training and artificial intelligence. These methodological associations have a high potential for applicability in the field [20], being potentially a faster and economic way to achieve correct and rapid diagnosis of the malaria species, even when health facilities receive more patients than their processing capacities and thus, becoming difficult to assist patients with a correct diagnosis [2, 11].

## Limitations of the study

Due to the limited format of the quantitative survey (41 questions), the number of images used was restricted, which has impacted the sensitivity values obtained in the study. A bigger survey with more images would be optimum but for this proof of concept addressed to non-expert people, more questions would have a negatively impact on the results. In order to obtain sufficient data, each species was represented by a 20%. As well, the limited number of stages showed for each species did not allow for a statistical analysis. This fact could have a high impact when some species forms are usually confounded with others (e.g. in *P. knowlesi*, 40% of the ring forms were confounded with *P. falciparum* rings). In order to reduce this impact and to achieve more realistic results, the proportion of the different stages was maintained as it appeared in the real samples. For *P. falciparum* and *P. knowlesi*, 80 and 50% of the images were rings respectively. In contrast, for *P. vivax* and *P. ovale* only 30% were rings and 40% were mature trophozoites. Finally, for *P. malariae* 50% of images were rings and 30% mature trophozoites. Thus, although some of these limitations should be resolved in the future, this work can be the used as the basis for designing a system and a protocol that allows for remote parasite classification in an operational context. Such system could use a mobile phone coupled to a regular microscope or directly a mobile microscope to digitalize the sample and transmit it to the internet over the mobile network. A specific app that is connected to a system could distribute the image samples to enough on-line examinators and produces an aggregate parasite classification that is sent back to the app in the field. The applications of such system could support directly in malaria diagnosis and quality control- but also could be used for training and educational purposes for students and field workers to improve the diagnosis performance.

## Conclusions

Using an on-line platform, it is possible to train on line non-expert volunteers to obtain an accurate diagnosis of *Plasmodium* species and stages, based on simple visual cues observed on parasitized red blood cells as texture, colour, shape and size. While the accuracy of a single on-line expert is far from perfect, a single parasite classification obtained by combining the opinions of multiple on-line volunteers over the same smear, could improve accuracy and reliability of *Plasmodium* species identification in remote malaria diagnosis.

## Additional file

**Additional file 1.** Example of supporting example images used in the query. (a) : young trophozoites, (b) mature trophozoites, (c) schizonts and (d) gametocytes of *Plasmodium knowlesi*. (e) Percentage of correct answers when support images were used (+) or not (−). Values given are the mean ± SEM calculated for the different images shown in a total of 32 volunteers. Asterisk indicates a significant difference between the percentage of response of the correct answer and each other two possibilities. **P* < 0.05.

## Abbreviations

ACT: artemisinin-based combination therapy
ANOVA: analysis of variance
CDC: Centers for Disease Control and Prevention
PCR: polymerase chain reaction
RBC: red blood cells
RDT: rapid diagnostic test
WHO: World Health Organization
