# Supplementary figures and images for: Plasmodium species differentiation by non-expert on-line volunteers for remote malaria field diagnosis
Source: Malar J. 2018 Jan 30;17:54. doi: 10.1186/s12936-018-2194-8 (PMC5789591; doi:10.1186/s12936-018-2194-8)

## Slide 1
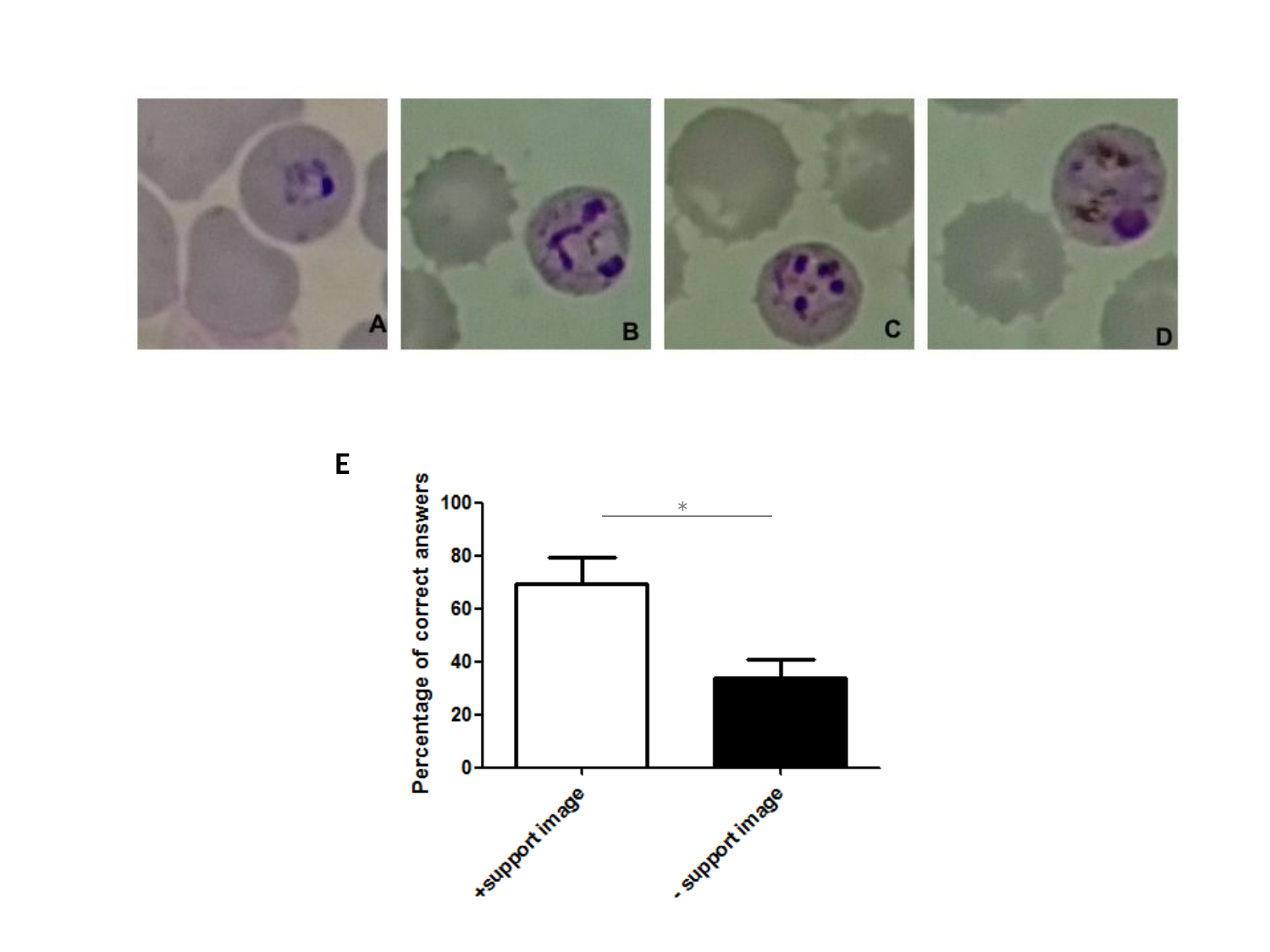

E
*

Supplement: Supplementary file 1 — Additional file 1. Example of supporting example images used in the query. (a) : young trophozoites, (b) mature trophozoites, (c) schizonts and (d) gametocytes of Plasmodium knowlesi. (e) Percentage of correct answers when support images were used (+) or not (−). Values given are the mean ± SEM calculated for the different images shown in a total of 32 volunteers. Asterisk indicates a significant difference between the percentage of response of the correct answer and each other two possibilities. *P < 0.05. [file 12936_2018_2194_MOESM1_ESM.pptx]
